# Medication safety in older adults in India: an integrative PhD synthesis of direct evidence and contextual implementation evidence

**DOI:** 10.1080/16549716.2026.2713867

**Published:** 2026-08-17

**Authors:** Saibal Das

**Affiliations:** a Department of Global Public Health, Karolinska Institutet, Stockholm, Sweden; b Indian Council of Medical Research - National Institute for Research in Bacterial Infections, Kolkata, India; c Academy of Scientific and Innovative Research, Ghaziabad, India

**Keywords:** Aging, deprescribing, India, informal healthcare providers, medication safety, polypharmacy, potentially inappropriate medication, self-medication

## Abstract

**Background:**

Unsafe medication practices among older adults are an important global health concern, particularly in low- and middle-income countries where multimorbidity, fragmented care, self-medication, and informal healthcare provision intersect.

**Objective(s):**

To synthesize direct evidence on medication safety among older adults in India and contextual evidence on deprescribing and community-level provider interventions relevant to safer medication use.

**Methods:**

This PhD synthesis integrates four studies: a record-based cross-sectional study on polypharmacy and cardiovascular autonomic function in Kolkata; a six-city community study of 600 Indian older adults; a systematic review and meta-analysis on deprescribing preventive medications in frail or end-of-life older adults; and a systematic review of informal healthcare provider interventions in low- and middle-income countries. Studies I–II provided direct Indian older-adult evidence, while Studies III–IV provided indirect contextual evidence for their optimization and implementation.

**Results:**

Polypharmacy was associated with higher anticholinergic burden and numerically higher cardiac autonomic neuropathy although residual confounding limits causal interpretation. In the multicity study, one-third had polypharmacy, while potentially inappropriate medications, prescribing omissions, and self-medication were common. Risks were higher with multimorbidity, recent hospitalization, care transitions, or living alone. Deprescribing showed no statistically significant increase in mortality, hospitalization, or major cardiovascular events, but heterogeneity was high and certainty low to very low. Informal-provider interventions showed the potential to improve knowledge, referral, case management, and medication-related practices.

**Conclusions:**

Medication safety among older adults in India requires an integrated continuum approach, but direct evidence supports only some components and implementation strategies that need prospective evaluation.

## Paper Context

- **Main findings:** Polypharmacy, potentially inappropriate prescribing, prescribing omissions, and self-medication were common among older adults in India, while supervised deprescribing and community-level provider interventions may improve medication safety.
- **Added knowledge:** This synthesis distinguishes direct Indian geriatric medication-safety evidence from indirect deprescribing and LMIC implementation evidence, and integrates them into a continuum model for safer medicine use.
- **Global health impact for policy and action:** Policies should prioritize structured medication review, deprescribing guidance, safer self-medication education, care-transition support, and pragmatic engagement of informal providers in community-based medication safety programs.

## Background

Population aging is reshaping health needs in India, where many older adults live with multimorbidity, and depend on long-term pharmacotherapy. While medicines are essential for managing chronic conditions, they can also cause preventable harm when used in excessive numbers, continued without reassessment, prescribed without attention to frailty or function or taken without adequate professional guidance. Age-related pharmacokinetic and pharmacodynamic changes, reduced physiological reserve, cognitive and sensory limitations, and caregiver dependence further increase medication-related risk [1–5]. The Indian context adds to the complexity. Older adults may consult multiple providers, move between hospitals and outpatient clinics, use prescriptions from different time points, purchase medicines directly from pharmacies, and combine allopathic medicines with traditional or complementary preparations. Medication lists are not always reconciled after discharge or specialist consultation, and prescription-only medicines may be obtained without a current prescription. Thus, medication safety is not only a prescribing issue but also a community, regulatory, behavioral, and health-system concern [3,6–13].

Polypharmacy, commonly defined as the concurrent use of five or more medicines, may be appropriate when each medicine has a clear indication and aligns with patient goals. However, inappropriate polypharmacy may arise from therapeutic duplication, prescribing cascades, drug–drug interactions, prolonged continuation of unnecessary medicines, or inadequate follow-up. Potentially inappropriate medications and potential prescribing omissions represent complementary forms of suboptimal pharmacotherapy: one reflects medicines where harms may exceed benefits, while the other reflects missed clinically indicated treatment [4–6]. Self-medication is another important pathway to harm, particularly among older adults using allopathic medicines or traditional preparations without adequate advice. Poor knowledge of dosing, duration, interactions, contraindications, adverse effects, and storage can compound this risk [7–10].

Deprescribing is equally important. In advanced frailty, dementia, or end-of-life contexts, the time to benefit from preventive medicines may exceed remaining life expectancy. Continuing antihypertensives, statins, anticoagulants, or antidiabetic agents without reassessment may increase hypotension, bleeding, hypoglycemia, falls, pill burden, cost, and reduced quality of life. Planned, monitored deprescribing aligned with goals of care is central to medication safety. Medication safety in India also intersects with informal healthcare providers, who are accessible, trusted, and embedded in communities across many low- and middle-income countries. Engaging them through structured, ethical, evidence-informed interventions may improve medicine use beyond formal facilities [14–21]. This PhD Synthesis condenses doctoral research examining medication safety among older adults in India across four connected levels: physiological consequences of medication burden, community patterns of unsafe medicine use, deprescribing as clinical optimization, and informal providers as community actors shaping medicine access and practice [14–17,22]. The overarching synthesis question was as follows: how evidence from patient-level medication burden, community medication practices, deprescribing research, and community-provider interventions be integrated to inform a pragmatic medication-safety framework for older adults in India?

## Methods

### Reporting approach and synthesis design

This PhD Synthesis integrates four doctoral studies: two observational cross-sectional studies and two systematic reviews. The reporting of the observational components was aligned with the Strengthening the Reporting of Observational Studies in Epidemiology (STROBE) checklist [23], and the reporting of the systematic-review components was aligned with the Preferred Reporting Items for Systematic Reviews and Meta-Analyses (PRISMA) 2020 statement. The purpose of this synthesis was not to pool all four studies statistically because they addressed complementary questions using different designs, populations, and outcomes. Instead, findings were integrated narratively across four domains: medication-related physiological vulnerability, community medication practices, deprescribing as clinical optimization, and informal healthcare providers as community-level actors [14–17, 22]. Table 1 summarizes the design, evidence base, reporting framework, and contributions of each doctoral study.

**Table 1.** Contribution of the four doctoral studies to this synthesis.

Study	Design and setting	Population/evidence base	Core contribution	Role in synthesis
Study I	Record-based cross-sectional study conducted in August 2022 at a community-based health consultation clinic for older adults in Kolkata, India	305 adults aged ≥ 60 years with chronic diseases and available cardiovascular autonomic function records	Linked polypharmacy and anticholinergic burden with cardiovascular autonomic vulnerability.	Direct Indian older-adult evidence
Study II	Six-city cross-sectional household study conducted in urban communities in Chennai, New Delhi, Kolkata, Ujjain, Guwahati, and Patna, India, from November 2022 to January 2024	600 community-dwelling adults aged ≥ 60 years selected through systematic random sampling	Estimated the burden and correlates of polypharmacy, PIMs, PPOs, and self-medication	Direct Indian older-adult evidence
Study III	Systematic review and meta-analysis	15 studies including > 33,000 participants with advanced frailty, dementia, or limited life expectancy	Synthesized evidence on deprescribing preventive medicines in frail or end-of-life older adults	Indirect clinical-context evidence for optimization and deprescribing
Study IV	Systematic review	38 intervention studies from low- and middle-income countries	Assessed interventions targeting informal healthcare providers and their relevance to community-level medication safety	Indirect LMIC implementation-context evidence

ACB: anticholinergic burden; LMICs: low- and middle-income countries; PIMs: potentially inappropriate medications; PPOs: potential prescribing omissions.

This article is an integrative PhD synthesis, not a formal systematic review of a single intervention or exposure. The synthesis used a structured narrative approach to integrate four doctoral studies according to a pre-specified conceptual logic: vulnerability, exposure, optimization, and system context. Vulnerability refers to aging, multimorbidity, frailty, autonomic dysfunction, cognitive limitations, and social isolation. Exposure refers to polypharmacy, anticholinergic burden, potentially inappropriate medications, prescribing omissions, traditional medicines, and self-medication. Optimization refers to medication review, reconciliation, prescribing appropriateness assessment, deprescribing, monitoring, and shared decision-making. System context refers to care transitions, pharmacies, informal providers, regulation, community trust, and access to formal care.

Studies I and II provide direct empirical evidence from older adults in India. Study III provides indirect clinical-context evidence on deprescribing in frail, end-of-life, or limited-life-expectancy older adults across settings. Study IV provides indirect implementation-context evidence on informal healthcare provider interventions in LMICs. These studies were therefore not treated as equivalent sources of direct evidence, but as complementary evidence streams.

### Study I: polypharmacy and cardiovascular autonomic function

Study I was a record-based cross-sectional study. Eligible participants were adults aged 60 years or older with chronic diseases who attended the clinic for medication review and reconciliation. Participants without documented cardiac autonomic function test records and those with cancer were excluded. All eligible records during the study period were considered, and no separate sampling procedures were applied [14,22]. The exposure of interest was, defined as the routine use of five or more oral allopathic medicines. Additional medication-related variables included the number of medicines, potentially inappropriate medications among participants with polypharmacy, and anticholinergic burden. Potentially inappropriate medications were identified using STOPP-START criteria, and anticholinergic burden was scored for each medicine and summed to produce a cumulative burden [4,14,24].

The main outcome was cardiac autonomic neuropathy, defined as an abnormality in at least one standardized cardiovascular autonomic function test. Tests included Valsalva ratio, orthostatic blood-pressure response, rise in diastolic blood pressure after isometric hand-grip exercise and heart-rate variability based on the expiration-to-inspiration ratio. Data sources were clinical records, medication histories, and autonomic test records. To reduce information bias, the study used predefined operational definitions and standardized test cut-offs [14,22]. Categorical variables were summarized as frequencies and percentages, and continuous variables as means and standard deviations. Between-group comparisons were performed for participants with and without polypharmacy. Binary logistic regression was used to estimate the associations between polypharmacy, anticholinergic burden, and cardiac autonomic neuropathy. The original regression models were adjusted for available demographic covariates. However, several clinically important cofounders, including diabetes duration, glycemic control, diabetic neuropathy, renal function, cardiovascular disease, frailty, BMI, orthostatic symptoms, antihypertensive class, and severity of multimorbidity, were not fully available or not comprehensively modeled. The results should therefore be interpreted as hypothesis-generating associations subject to residual confounding. Model fit was assessed in the original study using standard goodness-of-fit procedures. Results are reported as adjusted odds ratios (aOR) with 95% confidence intervals (95% CI) where available [14,22].

### Study II: community medication practices in six Indian cities

Study II was a cross-sectional household survey conducted between November 2022 and January 2024 in selected urban communities in six Indian cities: Chennai, New Delhi, Kolkata, Ujjain, Guwahati, and Patna. Households with adults aged 60 years or older were selected using systematic random sampling. Older adults unable to participate independently because of severe functional, cognitive, or hearing limitations were excluded [15,22]. The six cities were selected to represent geographically diverse urban Indian settings within the doctoral research network. Within each city, selected urban communities were surveyed using systematic household approaches. Each city contributed approximately equal numbers rather than population-weighted samples. When more than one eligible older adult was present, the eldest eligible person was selected. Non-response was handled by moving to the next eligible household according to the field protocol. Survey weights and design-effect adjustments were not applied; therefore, estimates should be interpreted as estimates from selected urban communities, not nationally representative Indian urban estimates. Trained study teams conducted door-to-door interviews using pre-tested questionnaires in the local languages. Data were collected on sociodemographic characteristics, comorbidities, hospitalization, and care transitions within the previous 3 months, prescriptions, current allopathic medicines, traditional and complementary medicines, and self-medication practices. Medical records and prescriptions were reviewed when available [15,22].

The primary outcome was the prevalence of polypharmacy, defined as current intake of five or more daily oral solid allopathic medicines for at least 4 weeks during the previous 3 months. Secondary outcomes included potentially inappropriate medications, potential prescribing omissions, self-medication, and knowledge, attitude, and reported practice related to self-medication. Potentially inappropriate medications and potential prescribing omissions were identified using STOPP-START version 3. Self-medication was defined as the use of over-the-counter or prescription medicines without a legitimate prescription by a registered medical practitioner, or the use of a prescribed medicine without following professional instructions for the present illness [15,25]. The estimated sample size was approximately 600, assuming 50% prevalence, 4% absolute precision, 95% confidence level, and allowance for field-level non-response. Descriptive analyses summarized participant characteristics and outcome prevalence. Binary logistic regression was used to identify factors associated with polypharmacy and self-medication. Candidate variables included age, sex, marital status, living arrangement, education, caregiver education, comorbidity burden, occupation, income, recent hospitalization, and recent care transition. Variables with *p* < 0.20 in univariable analysis were considered for multivariable models, and *p* < 0.05 was considered statistically significant in final models [15,22].

### Study III: deprescribing preventive medications

Study III was a systematic review and meta-analysis registered in PROSPERO (ID: CRD420251147086). Eligible studies were randomized controlled trials and observational comparative studies including adults aged 60 years or older with advanced frailty, dementia, or limited life expectancy in hospital, hospice, nursing-home, palliative-care, or community settings. Interventions were deprescribing, tapering, or de-intensification of preventive medications, including antihypertensives, statins, anticoagulants, and antidiabetics. Comparators were continuation or usual care. Studies without comparator groups, cross-sectional prevalence studies, feasibility studies, case reports, case series, editorials, qualitative studies, and preprints were excluded [16,26]. PubMed, Embase, Cochrane Library, Web of Science, CINAHL, and ProQuest Dissertations & Theses Global were searched from their inception to 3 October 2025 without language restriction. Search strategies combined controlled vocabulary and free-text terms for deprescribing, preventive medicines, frailty, dementia, limited life expectancy, and older adults. Records were de-duplicated, and titles/abstracts and full texts were screened independently by two reviewers, with disagreements resolved by discussion or senior review [16,26–29,30].

The primary outcome was all-cause mortality. Secondary outcomes included hospitalization, major adverse cardiovascular events, blood pressure, quality of life, falls, and fractures. Data extraction covered study design, setting, population, medication class, deprescribing strategy, comparator, follow-up, outcomes, and effect estimates. Risk of bias was assessed using RoB 2 for randomized trials and Risk Of Bias In Non-randomized Studies – of Interventions (ROBINS-I) for observational studies. Random-effects meta-analysis was performed when all studies were sufficiently comparable. Dichotomous outcomes were summarized as relative risks (RR) with 95% CIs; continuous outcomes were summarized as mean differences or standardized mean differences. Heterogeneity was assessed using the chi-square test and *I*^*2*^ statistic, and certainty of evidence was assessed using GRADE [16,27–29,30].

### Study IV: interventions targeting informal healthcare providers

Study IV was a systematic review registered in PROSPERO (ID: CRD42024521739). Eligible studies were original intervention studies from low- and middle-income countries that evaluated interventions targeting informal healthcare providers. Informal healthcare providers were defined as individuals lacking formal training recognized by national regulatory authorities, individuals not registered or regulated for practice, or individuals working privately outside a recognized scope of practice. Government-recognized community health workers were excluded [17,26]. PubMed, Cochrane CENTRAL, and Embase were searched for English-language studies published from 1990 to 30 June 2024. Titles/abstracts and full texts were screened by two independent reviewers, with disagreements resolved through discussion or an arbiter. Data were extracted on setting, provider type, intervention components, comparator, outcomes, and results.

Outcomes included knowledge, attitude, and reported practice; diagnosis and case management; referral; contraceptive use; and medication appropriateness [17,26]. Interventions were classified using the WHO health intervention classification framework as education and training, health services, policy or guideline interventions, and community-based interventions. Risk of bias was assessed using RoB 2 for randomized studies and ROBINS-I for quasi-experimental or non-randomized studies. Because of the heterogeneity in provider types, interventions, settings, and outcomes, findings were synthesized narratively. Certainty of evidence was assessed using GRADE [17,28,29].

### Ethical considerations

The two observational studies were performed in accordance with the Declaration of Helsinki and its subsequent amendments. Study I used de-identified record-based data and informed consent was waived by the relevant ethics committee. Study II obtained ethics approval from all participating sites, and written informed consent was obtained from participants or family members before recruitment and interview. Studies III and IV synthesized published data and did not require individual participant consent [14–17,22]. Ethics approval was not obtained from Karolinska Institutet, Stockholm, as no original data were collected in Sweden, and only analyzed data (without raw data containing patient identifiers) were shared from India with Karolinska Institutet, Stockholm.

## Results

### Study I: participant flow, descriptive data, and autonomic outcomes

In Study I, 321 older adults attended the clinic during the study period, and 305 eligible participants were included after applying the exclusion criteria. The mean age was 68.9 years, and 201 participants (65.9%) were male. Hypertension (81.9%) and type 2 diabetes mellitus (79.0%) were the most common comorbidities. Multimorbidity was frequent: 277 participants (90.8%) had at least two comorbidities, 219 (71.8%) had three comorbidities, and 103 (33.8%) had more than three [14,22]. Polypharmacy was present in 81 participants (26.6%). Among participants with polypharmacy, 42 individuals (51.8%) had 98 potentially inappropriate medications. Selected quantitative findings from the empirical studies are summarized in Table 2.

**Table 2.** Key findings and synthesis-level interpretations across the doctoral thesis.

Domain	Study	Key finding	Interpretation for the synthesis
Physiological vulnerability	Study I	CAN: 56.8% with polypharmacy vs 44.6% without polypharmacy; borderline/non-significant using stated denominators	Medication burden may mark autonomic vulnerability, but residual confounding and selected clinical sampling limit causal interpretation
Anticholinergic burden	Study I	Cumulative anticholinergic burden was higher with polypharmacy (8.1 ± 2.3 vs 2.3 ± 0.9)	Anticholinergic burden assessment may help identify older adults at greater risk from medication-related physiological harm
Community medication burden	Study II	Polypharmacy prevalence: 33.7% (95% confidence interval: 29.9–37.6%)	Substantial medicine burden exists among community-dwelling older adults across Indian urban settings
Medication quality	Study II	Potentially inappropriate medications: 28.8%; potential prescribing omissions: 20.3%	Overtreatment and undertreatment can coexist, requiring balanced medication optimization rather than medicine reduction alone
Self-medication	Study II	Self-medication prevalence: 19.7%; 65.3% had limited knowledge among those practicing self-medication	Unsafe medicine use is shaped by patient knowledge, access, and community medication practices
Deprescribing safety	Study III	No clear increase in mortality, hospitalization, or major adverse cardiovascular events; certainty very low	Deprescribing can be considered when aligned with goals of care, but requires monitoring and stronger evidence
Community implementation	Study IV	38 intervention studies from low- and middle-income countries; most were training-based or multifaceted; evidence certainty very low	Engagement and training of informal providers may support safer community-level care, but context-specific evaluation is needed

ACB: anticholinergic burden; CAN: cardiac autonomic neuropathy; CI: confidence interval; LMICs: low- and middle-income countries; MACE: major adverse cardiovascular events; PIMs: potentially inappropriate medications; PPOs: potential prescribing omissions.

Participants with polypharmacy had a higher cumulative anticholinergic burden than those without polypharmacy (8.1 ± 2.3 vs 2.3 ± 0.9; *p* = 0.03) and a higher mean anticholinergic burden score per anticholinergic medicine (3.0 ± 0.3 vs 1.6 ± 0.1; *p* = 0.03). Cardiac autonomic neuropathy was numerically more frequent among participants with polypharmacy than among those without polypharmacy (56.8% [46/81] vs 44.6% [100/224]); however, this comparison was borderline and not statistically significant using the stated denominator (uncorrected chi-square *p* ≈ 0.061; Fisher’s exact *p* ≈ 0.070) [14,22]. In adjusted analyses, polypharmacy was associated with higher odds of cardiac autonomic neuropathy compared with no polypharmacy (aOR: 2.66; 95% CI: 0.91–3.98). Compared with a cumulative anticholinergic burden of 0, a cumulative burden of 1–3 and >3 were also associated with higher odds of cardiac autonomic neuropathy (aOR: 1.60; 95% CI: 1.00–1.90; and aOR: 2.51; 95% CI: 0.99–3.52, respectively). These findings should be interpreted as associations because of the cross-sectional, record-based design [14,22].

### Study II: participant characteristics, community medication burden, self-medication, and medication literacy

Study II enrolled 600 community-dwelling older adults across six Indian cities. The mean age was 72.0 years, and 353 participants (58.8%) were women. Most participants were married (79.3%), lived with others (95.8%) and had secondary or lower education (81.1%). Three or more comorbidities were present in 423 participants (70.5%). Recent care transition within the previous 3 months was reported by 131 participants (21.8%), and hospitalization within the previous 3 months by 71 participants (11.8%) [15,22]. The overall prevalence of polypharmacy was 33.7% (95% CI: 29.9–37.6%). The number of daily oral solid allopathic medicines among participants with polypharmacy ranged from 5 to 18. Polypharmacy varied by city, with the highest prevalence in Guwahati and the lowest in Ujjain. In a multivariable analysis, polypharmacy was associated with three or more comorbidities (aOR: 2.5; 95% CI: 1.1–4.1), hospitalization within the previous 3 months (aOR: 4.6; 95% CI: 2.0–7.7) and care transition within the previous 3 months (aOR: 3.3; 95% CI: 1.4–5.7) [15,22].

Potentially inappropriate medications were identified in 173 participants (28.8%; 95% CI: 25.2–32.6%), and potential prescribing omissions in 122 participants (20.3%; 95% CI: 17.2–23.8%). Concurrent use of complementary or traditional medicines was reported by 164 participants (27.3%); among these, 80 participants (48.8%) used products without identifiable names or labels. The combined presence of potentially inappropriate medications and potential prescribing omissions indicates that medication quality problems include both overtreatment and undertreatment [15,22].

Self-medication was reported by 118 participants (19.7%; 95% CI: 16.6–23.1%). In the multivariable analysis, self-medication was independently associated with three or more comorbidities (aOR: 3.0; 95% CI: 1.4–6.7), hospitalization within the previous 3 months (aOR: 4.8; 95% CI: 1.5–8.0), and living alone (aOR: 4.5; 95% CI: 2.4–6.6). Among participants who practiced self-medication, 65.3% had limited knowledge, 50.0% were unaware of potential risks, and 40.7% reported unsafe self-medication behaviors [15,22]. These findings identify recent healthcare use, multimorbidity, and limited social support as important markers of medication-safety risk in the community. As with Study I, these associations should not be interpreted causally because the data were cross-sectional [15,22].

### Study III: study selection included evidence and deprescribing outcomes

Study III identified 10,397 records through database searches and included 15 studies with more than 33,000 participants after screening and eligibility assessment. Included studies evaluated the deprescribing, tapering, or de-intensification of preventive medicines among older adults with advanced frailty, dementia, or limited life expectancy. Medication classes included antihypertensives, statins, anticoagulants, and antidiabetics. Study settings included hospital, hospice, nursing-home, community, and palliative-care contexts [16,22].

Deprescribing was not associated with a clear increase in all-cause mortality (RR: 1.15; 95% CI: 0.98–1.35; *I*^*2*^ = 93%), hospitalization (RR: 0.93; 95% CI: 0.82–1.07; *I*^*2*^ = 68%), or major adverse cardiovascular events (RR: 1.37; 95% CI: 0.70–2.70; *I*^*2*^ = 95%). Deprescribing was also not associated with clear increases in fractures, falls, or deterioration in quality of life. Deprescribing antihypertensives was associated with a small increase in systolic blood pressure [16,22]. The certainty of evidence was very low for major outcomes because of the risk of bias, heterogeneity, imprecision, and clinical diversity across populations, interventions, and follow-up durations. Given high heterogeneity, risk of bias, imprecision, and very low certainty, these results should not be interpreted as evidence of equivalence, non-inferiority, or proven safety. Rather, current evidence is insufficient to rule out benefit or harm for several outcomes and medication classes [16,29].

### Study IV: study selection, intervention characteristics, and provider outcomes

Study IV screened 7,255 records and included 38 intervention studies from low- and middle-income countries. Informal healthcare providers included traditional healers, traditional birth attendants, medicine sellers, untrained allopathic providers, and other community-based providers outside formal regulatory recognition. Study contexts included maternal and child health, common infectious diseases, sexually transmitted infections, contraception, and medication use [17,22]. Education and training were the most common intervention components. Many interventions were multifaceted and combined training with supervision, referral mechanisms, health-service linkage, community activities, policy or guideline components, or supply-side support. Outcomes included diagnostic and case-management skills, knowledge, attitudes, reported practice, referral practices, contraceptive use, and medication-related practice [17,22].

Most studies reported improvement in at least one measured outcome; however, approximately one-quarter reported negative or no clear effects. Overall certainty of evidence was very low because of risk of bias, heterogeneity of interventions and outcome measures, and limited comparability across studies. The findings indicate that informal healthcare providers can be engaged to improve selected care practices, but isolated training is unlikely to be sufficient without supervision, referral pathways, and attention to legal and regulatory constraints [17,22,29].

## Discussion

### Principal synthesis

At the clinical-physiological level, polypharmacy and anticholinergic burden were associated with cardiac autonomic dysfunction. The synthesis is best interpreted as a continuum rather than as four equivalent studies addressing the same question. Studies I and II identify direct Indian older-adult medication-safety risks. Studies III and IV broaden the framework by informing how optimization and community implementation might be approached, while requiring cautious adaptation to the Indian geriatric context. The four studies together show that medication safety among older adults in India is a multi-level problem. At the community level, one in three older adults experienced polypharmacy, nearly one in three had potentially inappropriate medications, one in five had potential prescribing omissions, and one in five reported self-medication. At the optimization level, deprescribing preventive medications in frail or end-of-life populations did not show clear evidence of increased mortality, hospitalization, or major cardiovascular events, although certainty was very low. At the health-system level, interventions targeting informal providers may improve healthcare delivery practices but require careful design and sustained support. Table 3 translates this synthesis into intervention levels and implementation settings [14–17,22].

**Table 3.** Practical translation of the synthesis into a multi-level medication-safety intervention package.

Intervention level	Medication-safety problem	Evidence source	Evidence directness/certainty	Proposed action	Implementation safeguards	Research need
Patient and caregiver	Limited medication knowledge, unsafe self-medication, unlabelled traditional medicine use	Study II	Direct Indian evidence; cross-sectional	Medication literacy, counseling on OTC medicines, antibiotics, leftover medicines, and warning signs	Use local languages; involve caregivers; provide written medication lists	Test community medication-literacy interventions
Clinical encounter	Polypharmacy, PIMs, PPOs, anticholinergic burden	Studies I and II	Direct Indian evidence; observational	Structured medication review using STOPP-START, ACB assessment, indication review, and reconciliation	Avoid medicine-count-only decisions; consider multimorbidity and patient goals	Prospective trials of structured medication review
Care transition	Hospitalization and transitions associated with medicine duplication and self-medication	Study II	Direct Indian evidence; observational	Discharge medication list, early post-discharge review, duplicate removal, caregiver instructions	Prioritize older adults living alone or with multimorbidity	Evaluate transition-focused medication safety models
Frailty/end-of-life care	Preventive medicines with uncertain time-to-benefit	Study III	Indirect evidence; very low certainty	Shared deprescribing decisions based on prognosis, goals, adverse-effect risk, and monitoring	Do not infer universal safety; taper/monitor high-risk medicines	India-adapted deprescribing algorithms and trials
Digital/clinical decision support	Complex prescribing, interactions, PIMs, dose problems	External CDSS evidence	Indirect adjunct evidence	CDSS for PIM alerts, interaction checks, dose review, and reconciliation	Minimise alert fatigue; ensure usability and clinician adherence	Locally adapted CDSS evaluation in Indian geriatric care
Community health system	Reliance on informal providers and medicine sellers	Study IV	Indirect LMIC evidence; very low certainty	Training, supervision, referral pathways, rational medicine-use protocols	Engagement should not imply uncritical endorsement; align with regulation	Context-specific implementation studies

ACB: anticholinergic burden, OTC: over-the-counter, PIMs: potentially inappropriate medications, PPOs: potential prescribing omissions, STOPP-START: Screening Tool of Older Persons’ Prescriptions and Screening Tool to Alert to Right Treatment.

These results argue against a narrow interpretation of medication safety as simply reducing the number of medicines. The number of medicines is only one marker of risk. Some older adults need multiple medicines; others receive inappropriate medicines; some are missing indicated treatments; others take medicines without guidance; and some are exposed to advice and treatment from providers outside formal regulation. A safer system must therefore combine medication review, medication reconciliation, prescribing appropriateness assessment, deprescribing, patient education, caregiver support, pharmacy engagement, and community provider strategies [14–17,22].

### Polypharmacy as a marker of physiological risk

The association between polypharmacy, anticholinergic burden, and cardiac autonomic neuropathy is important because it links medication burden with measurable physiological vulnerability. Older adults are already at increased risk of orthostatic hypotension, impaired heart-rate variability, and fall. Medicines with anticholinergic or cardiovascular effects can further reduce autonomic adaptability. In resource-limited settings, autonomic testing may not be routinely available, but the clinical implications are clear: older adults taking multiple medicines, especially those with dizziness, falls, syncope, diabetes, hypertension, or high anticholinergic exposure, should be prioritized for medication review. Because diabetes and hypertension were highly prevalent and several clinical cofounders were incompletely captured, the observed association between polypharmacy, anticholinergic burden, and autonomic dysfunction should not be interpreted as independent causation. The findings also suggest that future medication safety interventions should include anticholinergic burden assessment. A simple medicine-count threshold may miss patients at high risk from a small number of high-burden medicines while also overidentifying patients whose polypharmacy is clinically justified. Combining count, indication, anticholinergic burden, fall risk, blood pressure instability, cognition, and patient priorities may provide a more clinically meaningful approach.

### Care transitions as critical windows

Study II identified recent hospitalization and care transitions as strong correlates of polypharmacy and self-medication. This is highly actionable. Hospital admission, discharge, specialist referral, emergency care and return to the community are moments when medicines are added, stopped, substituted, duplicated, or misunderstood. Older adults may retain pre-hospital medicines while adding discharge medicines, or may resume old prescriptions without clarifying whether they remain appropriate. These transitions are therefore critical windows for medication reconciliation. A practical response would include a structured medication review at discharge and within the first week after returning home. The review should identify all current medicines, including traditional preparations and medicines used without prescription; check indication, dose, duration, duplication, interactions, and adverse effects; identify potentially inappropriate medications and potential prescribing omissions; and provide a simple patient-held medication list. Community-level follow-up is especially important for older adults living alone, those with multimorbidity, and those recently hospitalized.

### PIMs and PPOs as two sides of medication quality

The simultaneous presence of potentially inappropriate medications and potential prescribing omissions shows that medication safety is not synonymous with fewer medicines. Older adults may be overtreated in one domain and undertreated in another. For example, an older adult may be taking medicine that increases fall risk while lacking an indicated preventive or symptom-relieving treatment. Medication optimization, therefore, requires clinical judgment rather than a blanket target to reduce the number of medicines. This is particularly relevant in India, where older adults may receive prescriptions from multiple providers, and may not have a single clinician responsible for the full medication list. Tools such as STOPP-START can support structured assessment, but they must be interpreted in light of local availability, patient preferences, goals of care, affordability, and monitoring capacity. The goal is not rigid adherence to criteria, but a disciplined process for identifying medicines that should be reviewed.

### Self-medication and limited awareness

Self-medication among older adults reflects both access gaps and behavioral realities. When formal care is costly, inconvenient, crowded, or difficult to reach, older adults may choose self-medication as a practical response. However, limited knowledge about dosing, adverse effects, interactions, and duration makes this practice risky. The finding that self-medication was associated with multimorbidity and recent hospitalization is particularly concerning because these individuals are likely to be taking several chronic medicines and may be more vulnerable to interactions and adverse effects. Medication safety interventions should therefore include patient and caregiver education. Education should not be limited to general warnings. It should provide concrete instructions: do not restart old antibiotics without medical advice; do not combine painkillers with kidney disease, anticoagulants, or uncontrolled hypertension without checking; keep an updated medication list; bring all medicines to every healthcare visit; ask which medicines were stopped or changed after discharge; and seek help for dizziness, confusion, falls, bleeding, or hypoglycemia. Pharmacy-level counseling and community outreach may be especially valuable.

### Deprescribing as patient-centred optimization

The deprescribing synthesis supports a cautious but positive interpretation. In frail or end-of-life older adults, deprescribing selected preventive medicines did not clearly increase major adverse outcomes. This does not mean deprescribing should be automatic. It means the continuation should not be automatic either. Preventive medicines should be reassessed when prognosis, frailty, goals, adverse effects, and treatment burden change. A patient-centred deprescribing process should begin with indication review and goal clarification. Medicines with limited near-term benefit, high adverse-effect risk, unclear indication, therapeutic duplication, or high burden should be prioritized for discussion [31]. Deprescribing should be supervised, gradual when necessary, and accompanied by monitoring plans and safety-netting. In India, where geriatric and palliative services are not uniformly available, deprescribing algorithms must be simple enough for primary care and community settings while retaining safeguards for high-risk medicines, such as anticoagulants, insulin, and antihypertensives. It is pertinent to mention here that for Study III, meta-regression was considered conceptually relevant for exploring heterogeneity by medication class, population, study design, setting, deprescribing approach, and follow-up duration. However, the small number of studies within several outcome-specific analyses and substantial clinical diversities limited the reliability of meta-regression.

### Informal healthcare providers and community medication safety

Informal healthcare providers influence medication use in many low- or middle-income countries’ communities. They are often closer to households than formal institutions and may be consulted before, after, or instead of formally trained practitioners. The systematic review shows that interventions involving informal providers can improve selected practices, but the evidence is inconsistent and very uncertain. This creates a policy dilemma ignoring informal providers is unrealistic, and uncritical legitimization may be unsafe. A pragmatic approach should distinguish engagement from endorsement. Engagement can include mapping informal providers, understanding common practices, training on danger signs and referral, discouraging high-risk practices such as irrational antibiotic use, improving record-keeping, and creating links with formal primary care. Interventions should be designed by regulators, professional bodies, community representatives, and formal healthcare providers. For older adults, training content should include high-risk medicines, polypharmacy, red flags after medication changes, avoidance of unnecessary antibiotics and injections, and referral pathways for suspected adverse drug reactions.

### A continuum model for safer medication use

**Figure 1.**
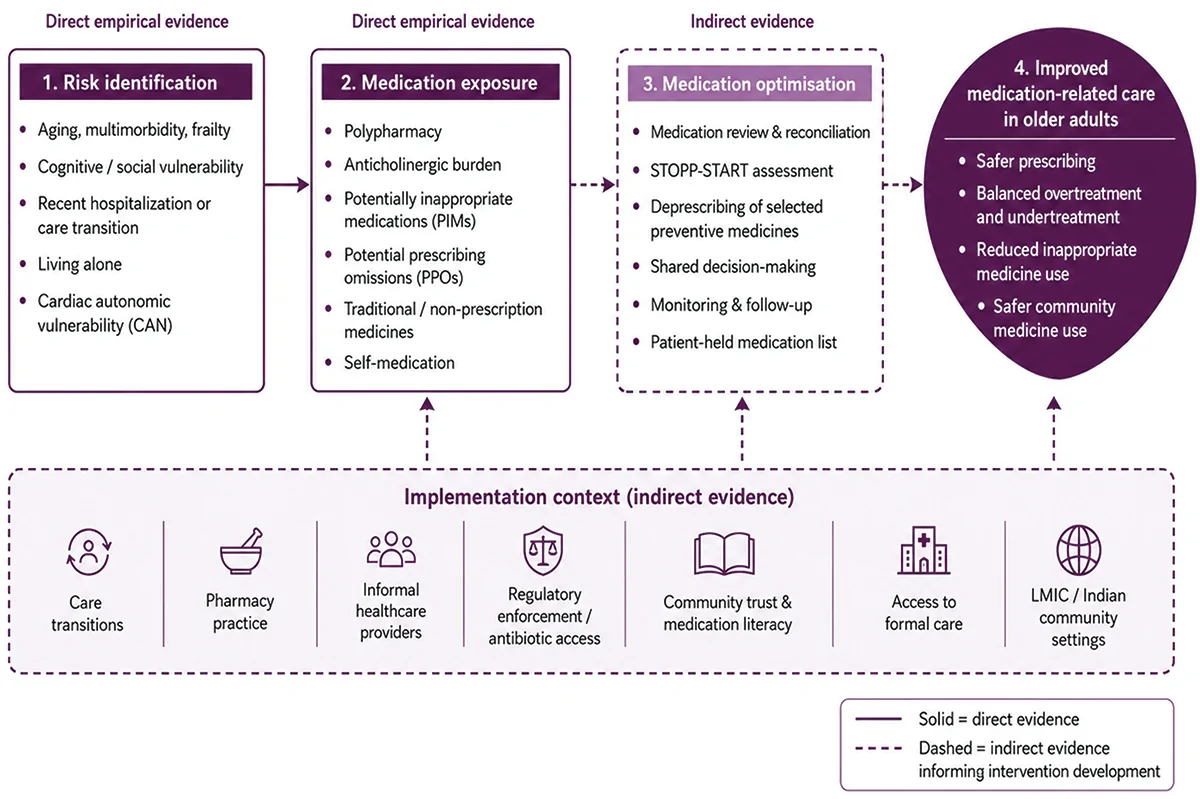
Logic model for improving medication-related care in older adults. The model distinguishes four linked domains: risk identification, medication exposure, medication optimisation, and implementation context. Risk identification and medication exposure are supported by direct empirical evidence from studies involving older adults in India. Medication optimisation and implementation-context components are informed by indirect evidence from deprescribing research and interventions involving informal healthcare providers in low- and middle-income countries. Solid borders and arrows indicate direct empirical evidence, whereas dashed borders and arrows indicate indirect evidence informing intervention development. The implementation context influences medication exposure, optimisation, and the achievement of safer medication-related care.

CAN, cardiac autonomic neuropathy; LMIC, low- and middle-income countries; PIMs, potentially inappropriate medications; PPOs, potential prescribing omissions; STOPP–START, Screening Tool of Older Persons’ Prescriptions and Screening Tool to Alert to Right Treatment.

The thesis supports a continuum model with four linked domains. The first domain is vulnerability: aging, multimorbidity, frailty, autonomic dysfunction, cognitive limitations, and social isolation. The second is exposure: polypharmacy, anticholinergic burden, potentially inappropriate medications, potential prescribing omissions, traditional medicines, and self-medication. The third is optimization: medication review, reconciliation, STOPP-START assessment, deprescribing, monitoring, shared decision-making, and patient-held medication lists. The fourth is system context: care transitions, pharmacy practice, informal providers, regulatory enforcement, community trust, and access to formal care. Interventions should connect these domains. For example, an older adult discharged after hospitalization should receive a reconciled medication list, anticholinergic and fall-risk review, screening for unnecessary preventive medicines, counseling against unsupervised medicine changes, and follow-up through primary care or community outreach. Informal providers and pharmacies should be oriented to refer rather than add medicines when older adults present with red-flag symptoms or complex regimens. Study IV is positioned at the base of the framework (Figure 1) because informal healthcare providers represent the broader community and health-system context within which medication-safety interventions are accessed, interpreted, and implemented, rather than a direct geriatric clinical intervention pathway. Figure 1 presents a logic model linking clinical and social vulnerabilities to medication exposure and subsequent medication optimization in older adults. Direct evidence informs risk identification and medication exposure, while indirect evidence supports optimization strategies, including medication review, deprescribing, and follow-up. The effectiveness of these interventions is influenced by the implementation context, including care transitions, pharmacy practice, informal healthcare providers, regulation, medication literacy, community trust, and access to formal healthcare.

### Strengths and limitations

The thesis has several strengths. It combines primary empirical data from Indian older adults with systematic reviews of clinical and health-system interventions. It addresses both overtreatment and undertreatment. It includes physiological outcomes, community behaviors, deprescribing evidence, and informal provider interventions, allowing a broader synthesis than any single study could provide. It also uses recognized tools, including STOPP-START, PRISMA, RoB 2, ROBINS-I, and GRADE. The limitations are also important. The observational studies were cross-sectional, so causal inference is limited. Study I was record-based and from one urban clinic; generalizability may be limited. Study II was conducted in selected urban communities and excluded older adults with severe functional, cognitive, or hearing limitations, who may have even greater medication-related vulnerability. Medication histories depended partly on participant reports and available documentation. Selecting the eldest eligible participant within households may have biased the sample toward older age, greater morbidity, and higher medication burden. Study III included clinically heterogeneous studies and produced very low-certainty evidence. Study IV also had very low-certainty evidence and diverse interventions, settings, and outcome measures. These limitations support cautious interpretation and point to the need for longitudinal, implementation-oriented research.

### Implications for policy, practice, and research

Medication safety should be integrated into India’s healthy ageing, non-communicable disease, antimicrobial resistance, and primary healthcare policies. National and state programmes for older adults should include medication review at care transitions, standard medication reconciliation tools, patient-held medication lists, monitoring of high-risk medicines, and clear guidance on self-medication. Regulation of over-the-counter access and antibiotic dispensing should be strengthened while ensuring that older adults can access legitimate care without excessive burden. Policies should also acknowledge the continued role of informal providers. Instead of relying only on punitive approaches, health systems should develop risk-reduction strategies that promote timely referral, discourage unsafe practices, and link informal providers with formal care pathways. These approaches require careful legal and ethical design, but are necessary in communities where informal providers are already embedded.

Clinicians should routinely ask older adults to bring all medicines, including traditional and non-prescription products, to consultations. Medication review should be prioritized after hospitalization or specialist visits, when five or more medicines are used, and when patients experience falls, dizziness, cognitive changes, multimorbidity, or social vulnerability. STOPP-START criteria can help structure reviews, but decisions should remain individualized [4,14,15,24,25]. Deprescribing should be framed not as withdrawal of care, but as active care that reduces harm, simplifies treatment, and aligns medicines with patient goals. Explicit monitoring plans are essential, particularly for blood pressure, glucose, anticoagulation, symptoms, and recurrence of disease indicators [16,22]. Clinical decision support systems may support drug-drug interaction checks, potentially inappropriate medication alerts, dose adjustment, anticholinergic burden review, and reconciliation. However, they should complement, not replace, clinical judgment, and require local adaptation to reduce alert fatigue, improve usability, and support adherence [32,33]. Future studies should evaluate longitudinal links between medication burden, anticholinergic burden, autonomic dysfunction, falls, hospitalization, and mortality in older Indian adults. Trials should test structured review and reconciliation aftercare transitions, while implementation studies should assess deprescribing models adapted to Indian primary care, geriatric, and palliative settings, including engagement of informal providers, pharmacies, and community health systems [14–17,22].

## Conclusions

This integrative synthesis indicates that polypharmacy, potentially inappropriate medications, prescribing omissions, and self-medication are common among older adults in selected urban Indian settings, with medication burden potentially reflecting physiological vulnerability. Indirect evidence from deprescribing and informal-provider interventions suggests possible optimization pathways, but certainty remains limited. Medication safety should therefore be strengthened through structured medication review, reconciliation at care transitions, medication literacy, supervised deprescribing, and context-sensitive community engagement, without overstating current evidence on safety or effectiveness.

## Supplementary Material

PRISMA.docx

STROBE.docx

## Data Availability

Full-text thesis link: https://doi.org/10.69622/30920123
